# Seasonal Dietary Composition of the Mountain Bongo (*Tragelaphus eurycerus* ssp. *isaaci*) at the Mount Kenya Wildlife Conservancy

**DOI:** 10.1002/ece3.74534

**Published:** 2026-10-27

**Authors:** Samuel Njuki Mahiga, Paul W. Webala, Dedan K. Ngatia, Robert Aruho, Joyce Omari, Evans M. Mwangi

**Affiliations:** ^1^ University of Nairobi Nairobi Kenya; ^2^ Mount Kenya Wildlife Conservancy Nanyuki Kenya; ^3^ Masai Mara University Narok Kenya; ^4^ Mpala Research Centre Nanyuki Kenya

**Keywords:** bongo, consumption, diet composition, feeding ecology, nutrition

## Abstract

The mountain bongo ( *Tragelaphus eurycerus* ssp. *isaaci*) represents a rare and critically endangered subspecies endemic to the Afromontane forests of Kenya. This study investigated the seasonal dietary composition of the mountain bongo at the Mount Kenya Wildlife Conservancy and the Mawingu Mountain Bongo Sanctuary. We conducted a two‐year observational study on the reintroduced wild population, employing random focal animal sampling at 30‐min intervals during active feeding periods from 06:00 to 11:00 and 16:00 to 19:00. Following each sampling session, we allocated 10 min to collect plant samples for subsequent identification. The data collected included the plant species consumed, the time spent on each species and the specific plant parts browsed. A total of 80 plant species, representing 43 families, were identified as components of the mountain bongo's diet, encompassing trees, shrubs, herbs, grasses, ferns, creepers, climbers and rhizomes. Seasonal variations exhibited minimal effects on herb or shrub consumption; however, conditions during the wet season were significantly correlated with a reduced likelihood of tree consumption. Seasonal dietary shifts, with reduced tree consumption during the wet season, underscore the importance of conserving a mosaic of vegetation types to ensure year‐round forage availability for mountain bongos.

## Graphical abstract

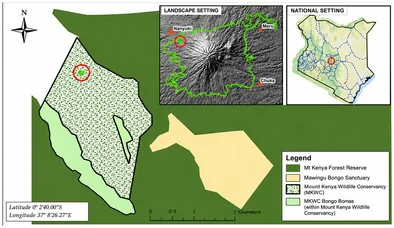

## 1 Introduction

The bongo, which weighs up to 400 kg, and belongs to the family Bovidae; Tragelaphinae. It is the only species in this particular subfamily where both sexes are characterised by spiral horns and a striking red chestnut‐coat colour, featuring white stripes that range from 9 to 16 in number on either side. As the largest and heaviest forest‐dwelling antelope in Africa, it exists in two subspecies: the mountain bongo (*Tragelaphus eurycerus isaaci*, Thomas, 1902), which is endemic to the Afromontane forests of Kenya, and the lowland bongo (*Tragelaphus eurycerus eurycerus*), found in West Africa (KWS 2026). Both subspecies are facing persistent threats from anthropogenic pressures, including indiscriminate snares that kill young and injure adults, as well as habitat loss due to human population growth and related economic activities causing habitat loss (Koopmans et al. 2021).

The mountain bongo is classified as critically endangered by the International Union for Conservation of Nature (IUCN 2016), and remains elusive within its endemic range (Faria et al. 2011; Fundi 2013; KWS 2019, 2026; Musyoki et al. 2012). The subspecies has a decreasing population trend, estimated at < 80 mature individuals in the wild (IUCN 2017, 2019; WRTI 2021). The national wildlife census reported the total remnant wild population as fewer than 100 individuals (Mahiga et al. 2026; Waweru et al. 2021). Historically, the species inhabited many of Kenya's montane forests, including the Aberdare Mountains, Cheranganyi Hills, Chepalungu, Mau, Mount Kenya and Mount Elgon forests. Today, the species occurs in severely fragmented and isolated populations, which are experiencing an extremely high risk of extinction. They commonly inhabit high‐latitude, rugged, thick montane forest between 2100 and 3000 m above sea level (Kingdon 1997; Svengren et al. 2017) in the Aberdare National Park, Mau Eburu National Forest Reserve, Maasai Mau Forest and Mount Kenya Forest Reserve (Fundi 2020; Kingdon 2015; KWS 2019, 2026; Svengren et al. 2017). The subspecies reaches sexual maturity at about 2 years of age, after which one offspring may be born every 2 years with a gestation period of 38 weeks (Kingdon 1997).

The decline in the wild populations began in the 1950s, primarily due to poaching (Estes et al. 2011; KWS 2019, 2026; Musyoki et al. 2012; Mwangi 2010) and habitat alteration (KWS 2026). Additional factors included predation by lions (Cheffings 1997; Prettejohn 2008) and a rinderpest outbreak that affected many other herbivore populations (Kock et al. 1999). The downward trend has resulted in local extinctions at Mt. Elgon, Cherangani and Chepalungu Hills (Altmann 1974; KWS 2019, 2026). Between 1966 and 1975, zoological institutions in the United States exported an estimated 36 wild mountain bongos from the Aberdare Forest. In the decades that followed, the wild population experienced a steady and alarming decline, prompting the initiation of a project in the 1990s aimed at repatriating mountain bongos. The Rare Species Conservatory Foundation (RSCF) spearheaded this effort in collaboration with the Mount Kenya Game Ranch (MKGR). The objective involved mobilising resources to facilitate the shipment of 18 bongos back to MKGR, where they would establish a breeding programme (Mahiga et al. 2026). This programme aimed to culminate in the reintroduction or supplementation of mountain bongos within their historical and current ranges. Subsequently, MKGR was rebranded as the Mount Kenya Wildlife Conservancy (MKWC).

The 2021 wildlife census report recommended the establishment of predator‐free sanctuaries and intensive management for the mountain bongo subspecies, aiming for a national population of 750 individuals over the next 50 years. This aligns with the targets set out in the National Recovery and Action Plan for the mountain bongo in Kenya for the period 2026–2030. The population at Mount Kenya Wildlife Conservancy (MKWC) offers an ideal context for studying the ecology of this subspecies. Griffith et al. (1989) highlighted that the re‐introduction of captive‐bred animal species could serve as a valuable strategy in addressing the global decline in biodiversity. In the case of the mountain bongo, the reintroduction of captive‐bred individuals represents an important step toward preventing the extinction of the subspecies (Fundi 2013; Musyoki et al. 2013).

Recovery consequently depends on captive breeding, the establishment of new breeding sanctuaries, and the eventual reintroduction and reinforcement of wild populations. Reintroduction decisions, however, rest on a detailed understanding of what the subspecies eats, where, and when—and precisely this understanding is lacking. Dietary composition and preference remain poorly understood for the mountain bongo (Fundi et al. 2022), and its feeding strategy is described as unusual and inadequately documented, with few evidence‐based captive feeding recommendations available (Wright et al. 2011).

The knowledge gap extends beyond species lists. Earlier work at the Mount Kenya Wildlife Conservancy showed that shrubs contribute approximately 55% of the diet and grass a further 27%, and that principal food plants are broadly represented at a proposed release site (72%–80% similarity in wet and dry seasons) (Fundi et al. 2022). That study, however, was restricted to male bongos, leaving sex‐ and age‐related variation in dietary composition unexamined. The seasonal dynamics of diet composition, particularly how the relative use of plant types shifts between wet and dry seasons and across sexes and age classes, therefore remain incompletely quantified.

To address this gap, the objective of this study was to investigate the seasonal diet composition of the endangered mountain bongo at the MKWC, thereby generating the ecological data needed to support the subspecies' recovery. Specifically, the study documented dietary composition during the wet and dry seasons to inform the selection of suitable sites for proposed breeding sanctuaries and release sites. The findings are intended to contribute to the National Recovery and Action Plan for Mountain Bongo 2026 to 2030, advancing its strategic goal of establishing two new breeding sanctuaries to supply animals for reintroduction and reinforcement of wild populations (KWS 2026). Characterising this dietary composition is essential for evaluating the nutritional viability of potential release habitats, as the species depends heavily on shrubs and grasses to meet its metabolic demands (Fundi et al. 2022).

## 2 Materials and Methods

### 2.1 Study Site

The study was conducted at the Mount Kenya Wildlife Conservancy (MKWC) and the adjacent Mawingu Mountain Bongo Sanctuary (MMBS), which encompass areas of 1250 and 776 acres, respectively (Figure 1). This region is located on the slopes of Mt. Kenya centred around latitude 0°2′40.00″ S and longitude 37°8′26.27″ E, with an altitude of 2387 m above sea level (Figure 1).

**FIGURE 1:**
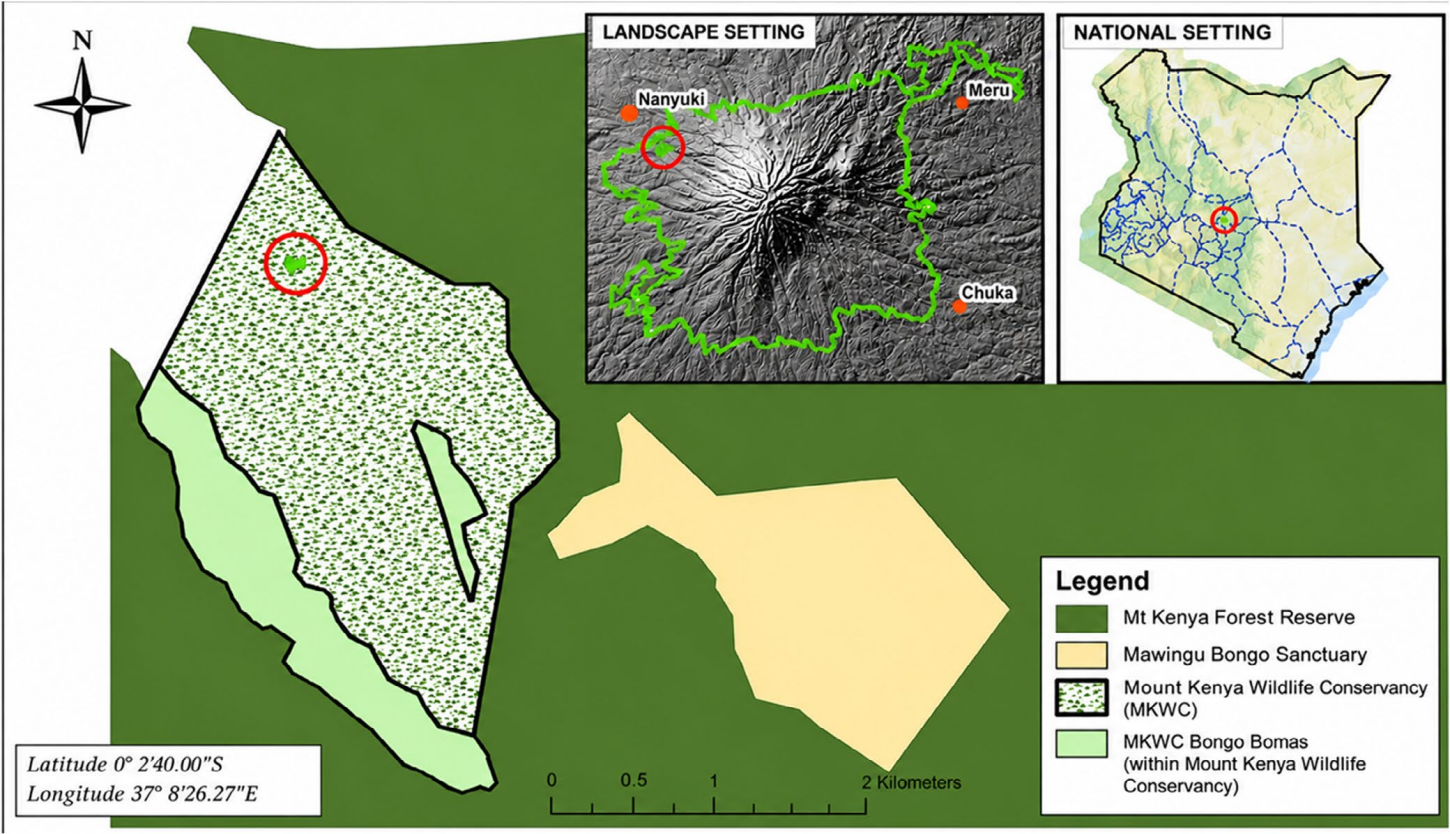
Map showing Mount Kenya Wildlife Conservancy (MKWC) and Mawingu Bongo Sanctuary (MMBS). Inset shows the study area location in Kenya.

Annual rainfall exhibits a bimodal pattern (Mahiga et al. 2026), characterised by two dry seasons and two wet seasons (Figure 2). The long rains occur between March and May, while the short rains take place from October to November.

**FIGURE 2:**
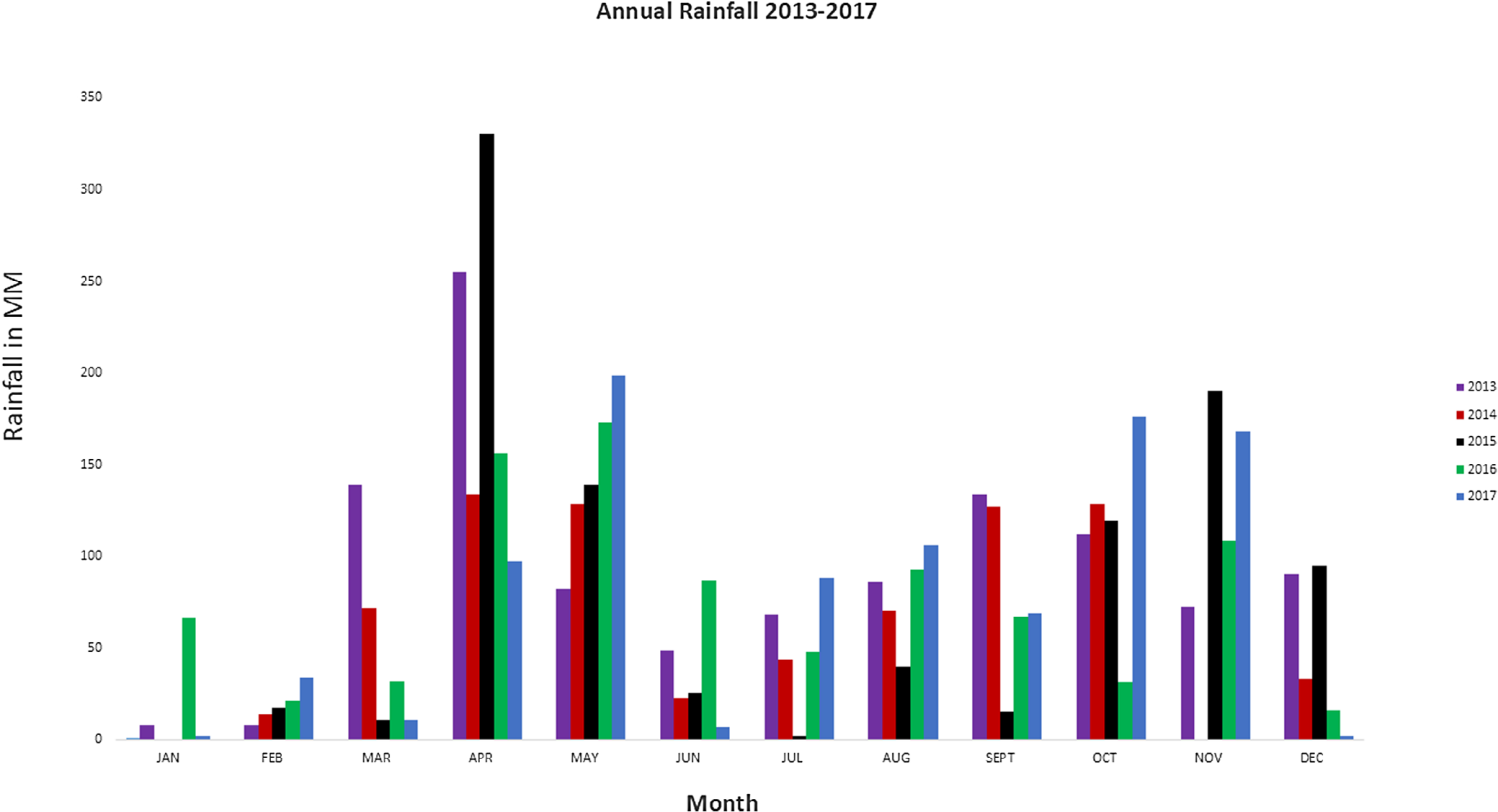
Rainfall trends 2012–2022 (William Holden Wildlife Foundation).

### 2.2 Field Methods

Data were collected from 62 Mountain Bongos, comprising two resident groups located at the two study sites within the research area. Following (Altmann 1974), random focal sampling was employed to study food selection. This process took place over twenty consecutive days each month during both the wet and dry seasons, spanning a two‐year period. Focal individuals were selected on a rotating basis, using their identification names. To facilitate random selection, name tags were created, with numbers assigned based on random tables. The tag numbers were shuffled using a computer each time prior to field sampling.

The focal animals were observed in 30‐min intervals during the periods of 06:00–11:00 and 16:00–19:00, when they were actively foraging. Data were recorded on the plant species being consumed by each focal individual, along with the duration spent on each species and the specific parts of the plant being browsed (including young leaves, mature leaves, shoots, flowers and fruits). This study categorised forage plants into four groups based on life forms and use by animals: grass, herbs, shrubs and trees. These categories are distinguished by their growth form, structural characteristics and the degree of woody tissue development.

### 2.3 Data Analysis

To examine how the diet composition of food‐plant type varied with season and the sex and age of bongo, we employed a Bayesian multinomial (categorical) mixed‐effects model. The response variable was the type of plant, modelled as a four‐category outcome (grass, herb, shrub and tree) using a multinomial logit link function. Separate linear predictors were estimated for each category in relation to a shared baseline structure. It is important to note that herbs encompass rhizomes and ferns, while shrubs include creepers and climbers.

The fixed effects included season and age class, which enabled us to assess their influence on the probability of selecting each plant type. To account for repeated observations from the same individuals, we included a random intercept for each individual (ID). The model was fitted to a total of 5310 bongo feeding observations, ensuring a robust estimation of both fixed and random effects while accounting for within‐individual variation in plant selection patterns. The reference category was implicitly represented through the multinomial logit framework. Model diagnostics indicated good convergence and reliable parameter estimation, with all Rhat values at 1.00 and high effective sample sizes. Data were analysed in *R* statistical software.

## 3 Results

A total of 80 plant species from 43 different plant families were recorded (Table 1) as being consumed by the mountain bongo. These species included trees, shrubs, herbs, grasses, ferns, creepers, climbers and rhizomes. Additionally, other food items observed being consumed included milk, mineral blocks and pellets.

**TABLE 1:** List of plant species consumed by bongos at MKWC and MMBS.

Species	Family	Frequency	Growth Form
*Abutilon mauritianum*	Malvaceae	96	Shrub
*Vachellia abyssinica*	Fabaceae	4	Tree
*V. tortilis*	Fabaceae	20	Tree
*Acalypha racemosa*	Euphorbiaceae	18	Shrub
*Achyranthes aspera*	Amaranthaceae	93	Herb
*Alchemilla fischeri*	Rosaceae	4	Herb
*Amaranthus graecizans*	Amaranthaceae	4	Herb
*A. retroflexus*	Amaranthaceae	4	Herb
*Amphicarpaea brachiata*	Fabaceae	2	Creeper
*Anthemis maritima*	Asteraceae	3	Herb
*Asparagus falcatus*	Asparagaceae	25	Climber
*Bidens pilosa*	Asteraceae	2	Herb
*Caesalpinia decapetala*	Fabaceae	6	Shrub/Climber
*Carissa spinarum*	Apocynaceae	32	Shrub
*Cenchrus ciliaris*	Poaceae	2	Grass
*Centella asiatica*	Apiaceae	17	Herb
*Clematis brachiata*	Ranunculaceae	3	Climber
*Commelina africana*	Commelinaceae	47	Herb
*Corymbia maculata*	Myrtaceae	17	Tree
*Crotalaria agatiflora*	Fabaceae	1	Shrub
*Cussonia holstii*	Araliaceae	13	Tree
*Cynanchum abyssinicum*	Apocynaceae	38	Climber
*Cynodon dactylon*	Poaceae	650	Grass (rhizomatous)
*Cyperus rigidifolius*	Cyperaceae	124	Rhizome (sedge)
*Cyphostemma kibweziense*	Vitaceae	35	Climber
*Dodonaea viscosa*	Sapindaceae	28	Shrub
*Dovyalis abyssinica*	Salicaceae	12	Shrub/Tree
*Dyschoriste radicans*	Acanthaceae	181	Herb
*Erica arborea*	Ericaceae	1	Shrub
*Erythrococca bongensis*	Euphorbiaceae	8	Shrub
*Euclea divinorum*	Ebenaceae	9	Shrub
*Galinsoga quadriradiata*	Asteraceae	22	Herb
*Galium aparine*	Rubiaceae	36	Creeper
*Geranium arabicum*	Geraniaceae	1	Herb
*Helichrysum italicum*	Asteraceae	9	Herb
*Hibiscus rosa‐sinensis*	Malvaceae	1	Shrub
*Hypoestes forskaolii*	Acanthaceae	133	Herb
*Indigofera arrecta*	Fabaceae	67	Shrub
*Indigofera swanziensis*	Fabaceae	2	Shrub
*Ipomoea purpurea*	Convolvulaceae	2	Climber
*Juniperus procera*	Cupressaceae	18	Tree
*Justicia diclipteroides*	Acanthaceae	51	Herb
*Lactuca canadensis*	Asteraceae	1	Herb
*Lannea cotoneaster*	Anacardiaceae	93	Tree
*Lantana trifolia*	Verbenaceae	58	Shrub
Lucerne ( *Medicago sativa* )	Fabaceae	878	Herb
*Maytenus heterophylla*	Celastraceae	99	Shrub
*Microglossa pyrifolia*	Asteraceae	6	Climber
*Mikania scandens*	Asteraceae	3	Climber
*Neonotonia wightii*	Fabaceae	50	Creeper
*Ocimum gratissimum*	Lamiaceae	23	Herb
*Oldenlandia monanthos*	Rubiaceae	2	Herb
*Olea africana*	Oleaceae	53	Tree
*Olinia rochetiana*	Oliniaceae	4	Tree
*Oxalis corniculata*	Oxalidaceae	1	Herb
*Oxalis griffithii*	Oxalidaceae	7	Herb
*Panicum monticola*	Poaceae	158	Grass
*Passiflora tripartita*	Passifloraceae	2	Climber
*Pavonia burchelli*	Malvaceae	1	Shrub
*Pennisetum clandestinum*	Poaceae	269	Grass
*Pentas lanceolata*	Rubiaceae	9	Shrub
*Periploca linearifolia*	Apocynaceae	21	Climber
*Phytolacca dodecandra*	Phytolaccaceae	5	Shrub
*Pilogyne scabra*	Cucurbitaceae	45	Creeper
*Pittosporum viridiflorum*	Pittosporaceae	10	Tree
*Plectranthus laxiflorus*	Lamiaceae	91	Herb
*Plectranthus sylvestris*	Lamiaceae	7	Herb
*Prunus africana*	Rosaceae	3	Tree
*Rhamnus prinoides*	Rhamnaceae	46	Shrub
*Rhus natalensis*	Anacardiaceae	423	Shrub
*Rubia cordifolia*	Rubiaceae	42	Climber
*Rubus niveus*	Rosaceae	26	Shrub
*Scutia myrtina*	Rhamnaceae	105	Shrub
*Senecio hadiensis*	Asteraceae	12	Herb
*Senecio* sp.	Asteraceae	6	Herb
*Sida tenuicarpa*	Malvaceae	30	Herb
*Solanum aculeastrum*	Solanaceae	68	Shrub
*S. mauense*	Solanaceae	35	Shrub
*S. mauritianum*	Solanaceae	7	Shrub
*Spermacoce princeae*	Rubiaceae	17	Herb
*Stephania abyssinica*	Menispermaceae	1	Climber
*Stipa keniensis*	Poaceae	1	Grass
*Tagetes minuta*	Asteraceae	3	Herb
*Toddalia asiatica*	Rutaceae	528	Climber
*Trichocladus ellipticus*	Hamamelidaceae	409	Tree
*Urtica massaica*	Urticaceae	42	Herb
*Vernonia adoensis*	Asteraceae	2	Shrub
*Warburgia ugandensis*	Canellaceae	43	Tree

### 3.1 The Influence of Season on the Diet Composition of Plant Types

There was a significant association between season and the type of food plants consumed (Figure 3; *p* < 0.01). However, the season appeared to have minimal influence on the consumption of herbs or shrubs, as the credible intervals overlapped zero. In contrast, conditions during the wet season were linked to a markedly lower probability of tree consumption (−0.76, 95% CI: −0.98 to −0.53), providing strong posteriori support for the existence of a seasonal effect.

**FIGURE 3:**
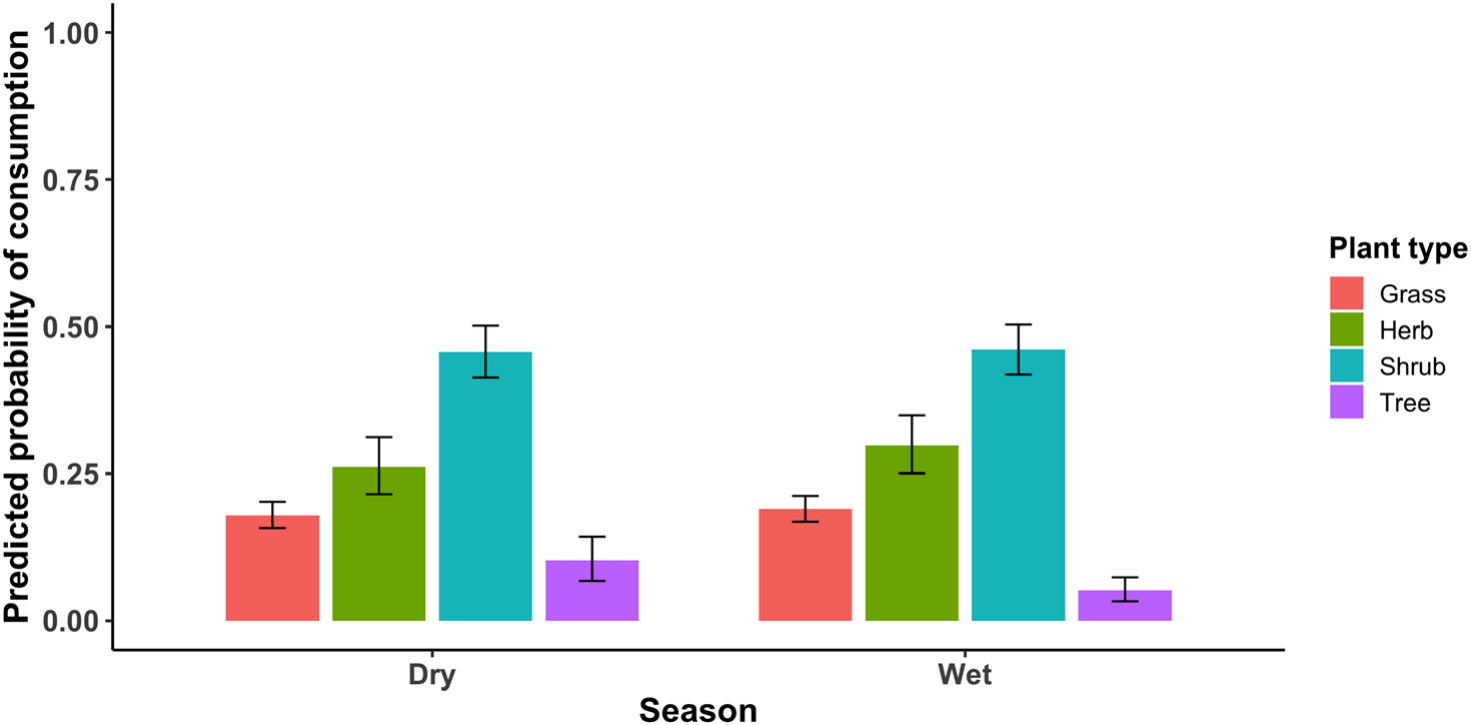
Influence of season on diet composition.

### 3.2 The Influence of Bongo Sex on Diet Composition

Similarly, there was a significant association between sex and food plant type (Figure 4; *p* < 0.01).

**FIGURE 4:**
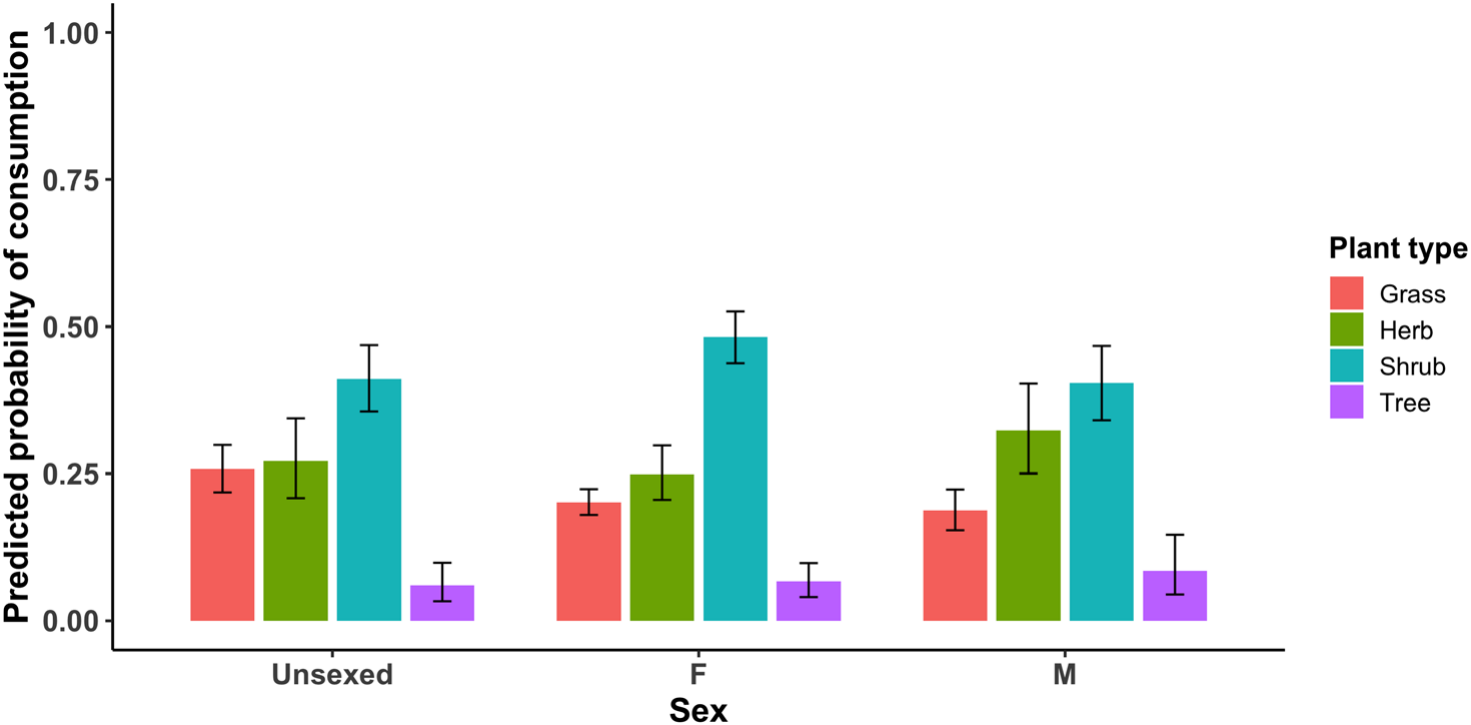
Influence of Bongo sex on diet composition.

### 3.3 Influence of Age of Bongo on Diet Composition

Age effects showed stronger patterns (Figure 5); calves were less likely to consume herbs (Estimate = −0.44, 95% CI: −0.87 to −0.01) and shrubs (Estimate = −0.46, 95% CI: −0.70 to −0.20) relative to the reference age class. Sub‐mature adults also showed reduced shrub consumption (Estimate = −0.30, 95% CI: −0.57 to −0.03), while their reduction in herb consumption was weaker and uncertain because the credible interval overlapped zero. Age effects on tree consumption were generally uncertain, with credible intervals overlapping zero for both calves and sub‐mature adults.

**FIGURE 5:**
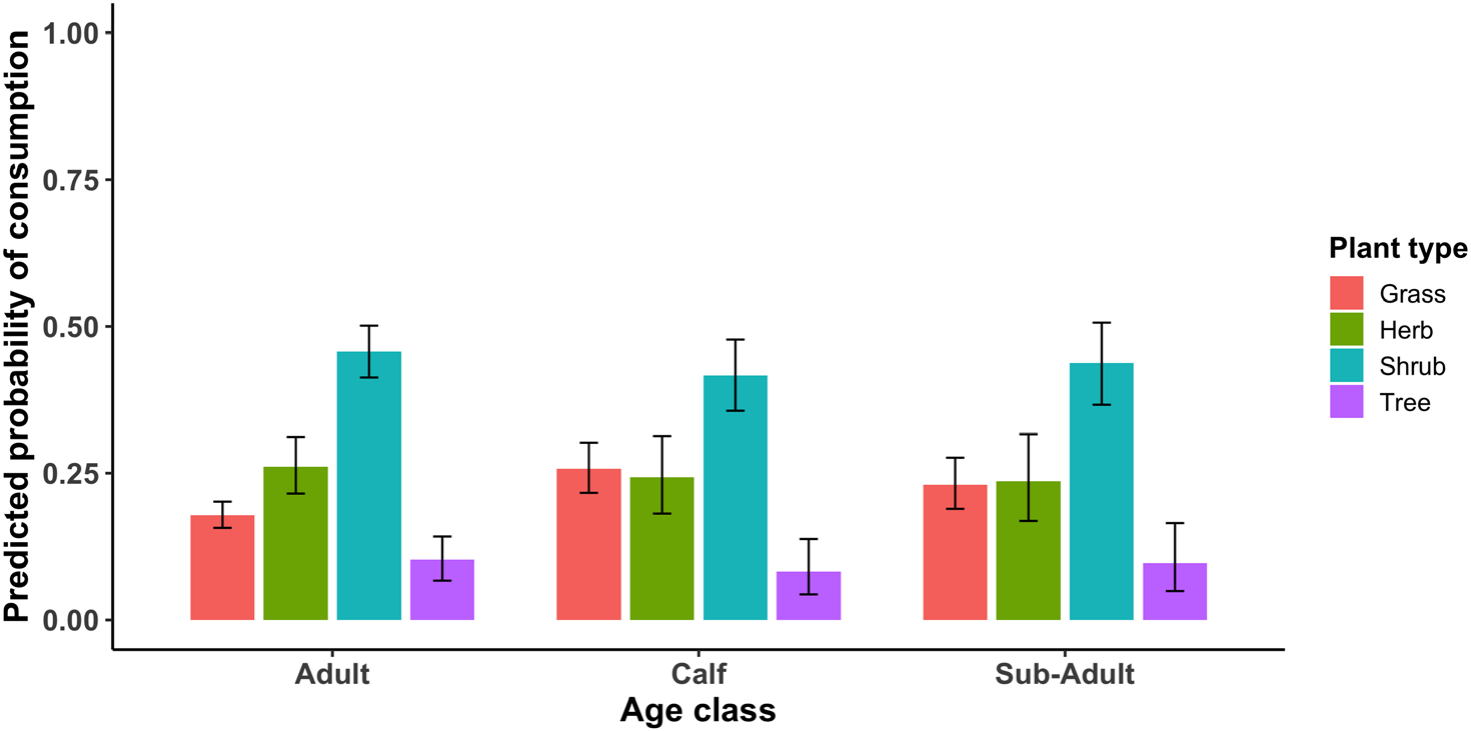
Influence of Bongo Age on diet composition.

Random intercept variation among individuals was moderate for herb consumption (SD = 0.64) and particularly high for tree consumption (SD = 1.08), suggesting substantial individual‐level differences in feeding preferences. Variation among individuals was comparatively smaller for shrub consumption (SD = 0.25). There was also a significant relationship between food type and age categories (Figure 4; *p* < 0.01).

## 4 Discussion

Our study reveals that the relative composition of plant species consumed by the mountain bongo varied seasonally. The use of herbs and shrubs remained consistent across seasons, whereas the contribution of trees declined significantly during the wet season. These patterns indicate that, although certain forage categories serve as year‐round staple resources, the mountain bongo also exploits tree‐derived forage opportunistically. Such dietary flexibility aligns with the adaptive foraging strategies reported for large herbivores in heterogeneous environments, in which animals track fluctuating resource patches (Littleford‐Colquhoun et al. 2024). Recent evidence from tropical forests similarly shows that dietary composition can differ markedly between dry and rainy seasons while a core set of plant taxa continues to be consumed (Aleixo‐Pais et al. 2023).

The dietary breadth documented here 80 plant species from 43 families is considerable for an African forest antelope and is consistent with the dicot dominated browsing diet typical of the Tragelaphini (Gagnon and Chew 2000). Comparable tragelaphines show narrower documented repertoires: bushbuck ( *Tragelaphus scriptus* ) in the Rwenzori National Park consumed 35 species, comprising three grasses, five shrubs and 27 herbs (Okiria 1980), and the mountain nyala ( *Tragelaphus buxtoni* ) in the Bale Mountains fed on 32 species from 18 families (Getachew and Girma 2022). Broad, generalised diets are likewise characteristic of forest herbivores beyond Africa; lowland tapirs ( *Tapirus terrestris* ) in a Neotropical rainforest consumed 61 plant species from 46 families (Lautenschlager et al. 2026). More broadly, tropical forest ruminants commonly ingest 50–80 fruit species in addition to foliage (Dubost 1984), and metabarcoding of African large‐herbivore assemblages indicates that wide food‐plant repertoires are widespread among tropical ungulates (Kartzinel et al. 2015; Pansu et al. 2022). Direct comparison is complicated by differences in sampling effort and method: the present study is based on 5310 focal animal feeding observations, whereas comparative studies have relied on stomach‐content analysis, scan sampling, or DNA metabarcoding. Nevertheless, the species count recorded here is comparable to local ecological knowledge identifying a broad suite of bongo browse species across the Kenyan forests (Sheppard et al. 2024), indicating that the mountain bongo is a broad generalist browser rather than a specialist feeder. Such breadth is ecologically significant because it buffers the population against seasonal shortfalls in any single forage category.

The stability of herb and shrub consumption across seasons suggests that these life forms form a foundational component of the bongo's dietary niche. In tropical forest ecosystems, understorey vegetation benefits from canopy‐mediated microclimatic buffering, which dampens thermal and hydrological variability and thereby stabilises primary productivity relative to more exposed vegetation strata (De Frenne et al. 2019). Comparable stability in the proportional use of woody plants and forbs across seasons has recently been documented in other browsing ungulates (Li et al. 2025), indicating that the retention of staple forage categories through the seasonal cycle is a widespread strategy among forest‐dwelling herbivores. Such consistency is critical for maintaining a dependable forage base and for minimising seasonal fluctuation in the resources available to herbivores.

In contrast, the marked decline in tree consumption during the wet season suggests a seasonal shift toward more ephemeral, higher‐quality resources (Klaus‐Hügi et al. 2000). This pattern accords closely with previous observations in the same population: at the Mount Kenya Wildlife Conservancy, *Trichocladus ellipticus*, despite its high relative abundance (1.88%), was less preferred during the wet season (E* = 0.20) yet was the most preferred species during the dry season (E* = 0.78), a seasonal reversal attributed to relative nutritive value (Fundi et al. 2022). Phenology‐driven shifts of this kind are well documented in other African browsing ruminants. Bushbuck favoured herbs over shrubs during the wet season (Okiria 1980), and kudu ( *Tragelaphus strepsiceros* ) switched from the leaves of palatable deciduous woody plants and forbs in the late wet season to evergreen foliage in the dry season, supplementing with newly flushed leaves of otherwise unpalatable woody species at the start of the growing season (Owen‐Smith and Cooper 1989). Comparable seasonal flexibility is now reported for tropical forest herbivores more widely: dietary composition differed markedly between wet and dry seasons in western red colobus in West Africa (Aleixo‐Pais et al. 2023), and analyses from seasonally dry tropical forests confirm systematic time‐dependence of forage quality, with the rainy‐season flush offering higher‐quality growth (Chankhao et al. 2026). Local ecological knowledge likewise indicates that bongo food choices differ with the seasons, former hunters describing that ‘bongo moved with the season, chasing the food they eat’ (Sheppard et al. 2024). Because rainfall stimulates a flush of succulent herbs and young shoots with lower lignin content and greater digestibility (Chebli et al. 2021), the reduced consumption of tree foliage during the wet season is consistent with tracking newly available, high‐quality understorey growth, while mature tree browse is retained as a fallback when such growth becomes scarce.

Conversely, tree resources are likely to function as critical fallback foods during dry periods. Fallback foods are characteristically consumed most heavily during periods of resource scarcity, and such dietary switching enables mixed‐feeding herbivores to buffer against seasonal shortfalls (Lambert and Rothman 2015). As understorey biomass declines and its quality deteriorates, the consumption of bark, shoots, and resilient tree foliage may supply the fibre and maintenance energy needed to survive periods of scarcity. This strategy is especially pertinent to large herbivores in montane habitats, where seasonal declines in forage quality can influence body condition and population performance, and it explains why tree browse may assume greater importance as preferred understorey resources dwindle.

In many tropical ecosystems, dry season forage is characterised by increased fibre content and lower crude protein concentrations, whereas the onset of rains restores higher‐quality growth. Under these conditions, tree‐derived leaves, shoots and bark commonly act as fallback foods that sustain herbivores when preferred forage is limited (Sensenig et al. 2010). The seasonal pattern documented here is therefore ecologically plausible and may represent an important survival strategy for the species.

From a conservation perspective, these findings carry direct management implications for both resident and reintroduced mountain bongo populations. To guarantee year‐round forage availability, habitat management should prioritise the preservation of structurally diverse forests containing a mosaic of herbs, shrubs and browsable tree species because tree forage may be most critical during nutritionally limiting dry periods. This recommendation is especially relevant for reintroduced populations occupying intensively managed or fenced landscapes, where forage diversity may be constrained (Fundi et al. 2022; Wright et al. 2011). Recent work on another critically endangered Kenyan antelope reinforces this point: restoration programmes have been urged to monitor and re‐establish preferred forage species and to provide supplementary diets in support of newly released herds (Ali and Kivai 2026). Incorporating such measures into sanctuary planning, alongside vegetation monitoring and local ecological knowledge of bongo browse species (Sheppard et al. 2024), would strengthen the evidence base for the National Recovery and Action Plan for Mountain Bongo.

The findings also underscore the importance of integrating seasonality into nutritional monitoring and supplementary feeding plans. During the dry season, managers may need to improve access to browse material or provide targeted supplementation during periods of low forage productivity. In small and recovering populations, such interventions could improve body condition, reproductive success and juvenile survival, particularly given that the use of fallback foods and seasonal dietary switching are associated with fitness proxies and population performance in endangered herbivores (Harvey Sky et al. 2024).

Although informative, these findings should be interpreted alongside several limitations. Plant categories were grouped broadly into grass, herbs, shrubs and trees, which may mask species‐level differences in use and quality among forage taxa. Seasonal effects may also vary with rainfall intensity, habitat condition, reproductive status and local population density, and the extent of seasonal diet switching in large herbivores is known to be modulated by body size and mobility (Littleford‐Colquhoun et al. 2024). Future studies should integrate botanical inventories, nutrient analyses, vegetation phenology and behavioural time budgets to clarify the mechanisms underlying seasonal diet shifts.

## 5 Conclusion

It is imperative to comprehend the seasonal grazing dynamics in order to achieve long‐term population recovery, reintroduction success and evidence‐based habitat management (Fundi et al. 2022; Kenya Wildlife Service 2019). In our study, the mountain Bongo demonstrated a seasonal adjustment in its forage utilisation. This is demonstrated by its comparatively consistent use of herbs and shrubs and reduced tree consumption during the wet season. These findings highlight dietary plasticity and emphasise the importance of diverse vegetation structure in supporting this critically endangered antelope. Understanding these seasonal feeding dynamics is essential for evidence‐based habitat management, the success of reintroduction efforts and the long‐term recovery of the population (Fundi et al. 2022; KWS 2019).

## Author Contributions

**Samuel Njuki Mahiga:** conceptualization (lead), data curation (lead), formal analysis (lead), funding acquisition (lead), investigation (lead), methodology (lead), project administration (lead), resources (lead), validation (lead), visualization (lead), writing – original draft (lead), writing – review and editing (lead). **Paul W. Webala:** conceptualization (equal), data curation (equal), formal analysis (equal), funding acquisition (equal), investigation (equal), methodology (equal), project administration (equal), resources (equal), supervision (lead), validation (equal), visualization (equal), writing – original draft (equal), writing – review and editing (equal). **Dedan K. Ngatia:** formal analysis (supporting), methodology (equal), validation (equal). **Robert Aruho:** conceptualization (equal), funding acquisition (lead), investigation (equal), methodology (equal), project administration (equal), resources (equal), writing – original draft (equal), writing – review and editing (equal). **Joyce Omari:** conceptualization (supporting), data curation (supporting), formal analysis (supporting), funding acquisition (supporting), investigation (equal), methodology (supporting), supervision (lead), writing – original draft (supporting), writing – review and editing (supporting). **Evans M. Mwangi:** conceptualization (lead), data curation (lead), formal analysis (lead), funding acquisition (lead), investigation (lead), supervision (lead), validation (lead), writing – original draft (supporting), writing – review and editing (supporting).

## Funding

This work was supported by Rufford Foundation (38463‐1), Mount Kenya Wildlife Conservancy (2022) and Wildlife Conservation Network (WCN) (2022).

## Conflicts of Interest

The authors declare no conflicts of interest.

## Supporting information


**Data S1:** ece374534‐sup‐0001‐Supinfo1.R.

**Data S2:** ece374534‐sup‐0002‐Supinfo2.csv.

## Data Availability

We have provided the all‐data files for publication and as Supporting Information.
